# Clinical evaluation of Dachai Wendan Decoction in the treatment of metabolic syndrome induced by atypical antipsychotics: A retrospective study

**DOI:** 10.1097/MD.0000000000046908

**Published:** 2026-03-20

**Authors:** Yuze Wu, Xia Zhao, Jiefeng Cui, Menghan Lv, Bo Hu, Chunyan Xu, Junran Zheng, Fude Yang, Shaoxiao Yan

**Affiliations:** a Department of Integrated Chinese and Western Medicine, Beijing Huilongguan Hospital, Beijing, China.

**Keywords:** atypical antipsychotics, complementary and integrative medicine, Dachai Wendan Decoction, metabolic syndrome, schizophrenia

## Abstract

This retrospective study evaluated the clinical effectiveness and safety of Dachai Wendan Decoction (DWD) in combination with standard Western Medicine (WM) for the management of metabolic syndrome (MetS) induced by atypical antipsychotic medications. A total of 107 patients diagnosed with schizophrenia according to the International Classification of Diseases, Tenth Revision, and fulfilling the International Diabetes Federation criteria for MetS, were included between January 2020 and December 2024. Patients were allocated to either the WM group (n = 56) or the integrative medicine group receiving DWD in addition to WM (DWD + WM, n = 51). After 8 weeks of treatment, the remission rate of MetS was significantly higher in the DWD + WM group (47.1%) compared with the WM group (25.0%, *P* = .02). The DWD + WM group demonstrated greater reductions in body weight, body mass index, waist circumference, and blood pressure. Improvements in lipid metabolism were also more pronounced, with greater decreases in triglycerides, total cholesterol, and low-density lipoprotein cholesterol, alongside an increase in high-density lipoprotein cholesterol. Glycemic control, reflected by fasting plasma glucose and glycated hemoglobin, also improved significantly. Psychiatric symptoms remained stable in both groups, with no dosage adjustments of antipsychotics required. Both treatments were well tolerated, and the incidence of adverse events was low and comparable. These findings indicate that adjunctive therapy with DWD is a safe and effective strategy to improve metabolic outcomes in patients with antipsychotic-induced MetS.

## 1. Introduction

The advent of second‐generation (atypical) antipsychotics (AAPs) has markedly improved the management of schizophrenia and other psychotic disorders by reducing extrapyramidal side effects and addressing negative symptoms. However, their metabolic side effects, including weight gain, dyslipidemia, insulin resistance, and hypertension, have emerged as a major limiting factor in long-term treatment. Atypical antipsychotic therapy is associated with a threefold increase in risk of metabolic syndrome (MetS), type 2 diabetes mellitus, and cardiovascular morbidity relative to the general population.^[1]^ Early identification and intervention for metabolic disturbances in patients receiving AAPs are now considered essential to reduce morbidity and mortality. Clinical guidelines recommend regular metabolic monitoring – at baseline, 4 months after initiation or change of antipsychotic, and annually thereafter – including measurement of weight, waist circumference, fasting glucose, and lipids. Despite these guidelines, adherence to metabolic surveillance remains suboptimal in many psychiatric settings, delaying detection and intervention.^[2]^ Meanwhile, pharmacologic strategies for antipsychotic-induced metabolic syndrome (AIMS) beyond metformin remain limited. Systematic reviews suggest that augmentation with glucagon-like peptide-1 (GLP-1) receptor agonists, statins, or fibrates may yield benefit, but evidence in psychiatric populations remains sparse and heterogeneous.^[3]^

In parallel, Traditional Chinese Medicine (TCM) has been explored as adjunctive therapy to ameliorate antipsychotic side effects, particularly metabolic dysregulation. A recent systematic review of herbal medicine add-on to antipsychotics in psychiatric populations demonstrated modest improvement in lipid profiles, glucose parameters, and body weight, though included trials often lacked metabolic endpoints as primary outcomes.^[4,5]^ In the general (non-psychiatric) MetS population, meta-analyses suggest that TCM combined with conventional therapy may produce superior composite remission of MetS and enhancement in lipid/glucose parameters compared to Western Medicine (WM) alone, albeit with heterogeneity and variable methodological quality. Furthermore, recent advances in metabolomics and spatial metabolomics provide mechanistic insight into how multi-component herbal formulas may modulate metabolic and inflammatory pathways in a tissue-specific manner, offering novel translational support for TCM interventions.^[6]^ Dachai Wendan Decoction (DWD), a TCM formula combining Wendan Tang and Xiao Chaihu Tang elements, is hypothesized to harmonize liver and gallbladder, resolve “phlegm-heat,” and restore Qi–water balance in classical theory. Biomedically, its constituent herbs may influence bile acid metabolism, intestinal motility, and lipid mobilization. However, clinical evidence of DWD in AIMS remains lacking.

Given the unmet need for effective, well-tolerated adjunctive strategies, we conducted a retrospective clinical study comparing DWD + standard Western metabolic therapy (metformin, statins, lifestyle) versus Western therapy alone in patients with AIMS, with the primary endpoint of MetS remission at 8 weeks. The findings may help inform integrated care strategies in patients receiving antipsychotics, balancing metabolic risk mitigation and psychiatric stability.

## 2. Methods

### 2.1. Study design

This study was approved by the Ethics Committee of Beijing Huilongguan Hospital and conducted in accordance with the Declaration of Helsinki. As a retrospective study, all eligible patients who met the diagnostic criteria were consecutively identified from the hospital electronic medical record system between January 2020 and December 2024, in order to minimize selection bias. Patients were included if they met the International Classification of Diseases, Tenth Revision (ICD-10) diagnostic criteria for schizophrenia and fulfilled the International Diabetes Federation (IDF) criteria for MetS that developed after atypical antipsychotic treatment.

To reduce potential confounding, only patients who had been receiving AAPs at a stable dose for at least 3 months, with stable psychiatric symptoms and no dosage adjustments, were enrolled. Baseline demographic, psychiatric, and metabolic characteristics were carefully reviewed, and no significant differences were found between the 2 groups.

The inclusion criteria were: age between 18 and 65 years and stable antipsychotic treatment as described above. Exclusion criteria were applied to avoid factors that could independently affect metabolic outcomes and included: impulsive, suicidal, or self-injurious behavior; severe hepatic or renal impairment, cardiac insufficiency, acute complications, or severe infections; comorbid substance or alcohol abuse; endocrine disorders (e.g., Cushing syndrome); central obesity caused by long-term corticosteroid use; type 1 diabetes mellitus, secondary hypertension, or familial dyslipidemia; known allergy to herbal medicine components; or other clinically significant laboratory abnormalities unrelated to MetS.

After eligibility assessment, patients were assigned to either the WM group (n = 56) or the integrative medicine group receiving DWD in addition to WM (DWD + WM, n = 51), according to the treatment they received in clinical practice. Because treatment allocation was based on routine clinical decisions rather than randomization, careful baseline comparison was conducted to ensure group comparability and reduce confounding. Written informed consent was obtained from all participants or their legal guardians.

### 2.2. Diagnostic criteria

The diagnosis of schizophrenia was established according to the ICD-10. Patients were required to present with at least one of the following characteristic symptoms, or with 2 if less specific manifestations were observed, persisting for a minimum of one month: persistent delusions that are culturally inappropriate and completely impossible (e.g., delusions of control, thought insertion, withdrawal, or broadcasting); persistent auditory hallucinations, particularly voices giving a running commentary on the patient’s actions or voices conversing among themselves; disorganized thought processes manifesting as incoherence, irrelevant speech, or neologisms; or catatonic behaviors such as stupor, mutism, posturing, or excitement. In the absence of these core symptoms, the diagnosis could be made when at least 2 of the following sustained features were present: persistent hallucinations of any modality accompanied by fleeting or half-formed delusions without clear affective content, thought breaks resulting in incoherence, less prominent catatonic phenomena, persistent negative symptoms such as blunted affect, apathy, or social withdrawal not attributable to depression or medication effects, and marked changes in personal behavior such as loss of interest or self-neglect. These symptoms were required to produce significant impairment in daily functioning and not to be explained by mood disorders, substance use, or organic brain disease.

The diagnosis of MetS was made in accordance with the criteria of the IDF (IDF, 2005), with attribution to atypical antipsychotic use determined by temporal association. A diagnosis required central obesity, defined by ethnic-specific waist circumference thresholds (≥90 cm for men and ≥80 cm for women in Asian populations), in addition to at least 2 of the following conditions: elevated triglycerides (≥150 mg/dL or 1.7 mmol/L, or treatment for hypertriglyceridemia); reduced high-density lipoprotein cholesterol (HDL-C) (<40 mg/dL or 1.03 mmol/L in men, <50 mg/dL or 1.29 mmol/L in women, or treatment for low HDL-C); elevated blood pressure (systolic ≥ 130 mm Hg or diastolic ≥ 85 mm Hg, or treatment of previously diagnosed hypertension); and impaired fasting glucose (≥100 mg/dL or 5.6 mmol/L, or previously diagnosed type 2 diabetes mellitus). MetS was considered atypical antipsychotic–induced if the metabolic abnormalities emerged after the initiation of antipsychotic therapy in patients without preexisting metabolic disease, or if preexisting metabolic disturbances demonstrated significant aggravation temporally related to antipsychotic exposure.

### 2.3. Intervention protocols

#### 2.3.1. WM group (WM)

Patients in the WM group received standard metabolic management while maintaining a stable atypical antipsychotic regimen. Interventions included structured lifestyle modification – comprising dietary counseling, exercise recommendations, and health education – along with pharmacotherapy according to individual metabolic abnormalities. Metformin was prescribed as the first-line agent for impaired glucose regulation, statins or fibrates were administered for dyslipidemia, and antihypertensive drugs were used as clinically indicated. All treatments were consistent with contemporary clinical guidelines, and metabolic parameters were monitored regularly during follow-up.

#### 2.3.2. Integrative medicine group (DWD + WM)

Patients in the integrative medicine group received the same WM management protocol described above, in combination with DWD. The decoction was prepared according to a standardized formula in the hospital’s TCM pharmacy, using herbs sourced from a single GMP-certified supplier. The core prescription consisted of Bupleuri Radix, Scutellariae Radix, Pinelliae Rhizoma preparatum, Bambusae Caulis in taeniam, Aurantii Fructus immaturus, Citri Reticulatae Pericarpium, Poria, Glycyrrhizae Radix, Zingiberis Rhizoma recens, and Jujubae Fructus, yielding approximately 200 to 250 mL per dose, administered twice daily after meals for at least 8 weeks. Minor symptom-based adjustments (addition or removal of 1–2 herbs) were permitted without altering the principal components.

Adherence to the herbal treatment was assessed using pharmacy dispensing records and medical documentation. Patients who completed ≥80% of the prescribed doses were considered adherent. Adverse events were recorded throughout the treatment period.

### 2.4. Data collection

Clinical and laboratory data were retrospectively extracted from the electronic medical records of all eligible patients. Baseline demographic and clinical variables included sex, age, duration of psychiatric illness, subtype of schizophrenia, and type and dosage of AAPs. Baseline metabolic indicators comprised body weight, body mass index (BMI), waist circumference, systolic and diastolic blood pressure, fasting plasma glucose, glycated hemoglobin (HbA1c), triglycerides, HDL-C, low-density lipoprotein cholesterol (LDL-C), and total cholesterol. Follow-up data were collected at 8 weeks after initiation of treatment. The primary outcome measures were the remission rate of MetS, defined according to the IDF criteria, changes in body weight, BMI, waist circumference, and blood pressure. Secondary outcomes included changes in lipid parameters (triglycerides, HDL-C, LDL-C, and total cholesterol), glycemic indicators (fasting plasma glucose and HbA1c), and psychiatric symptom stability, which was assessed using the Positive and Negative Syndrome Scale (PANSS) scores and documentation of any antipsychotic dose adjustments. Safety outcomes were evaluated by reviewing adverse events documented during the treatment period, including gastrointestinal discomfort, allergic reactions, liver transaminase elevations, and renal function abnormalities. Treatment adherence was assessed based on medication records and physician notes, with adherence defined as completion of ≥ 80% of the prescribed regimen.

### 2.5. Statistical analysis

Statistical analyses were performed using Statistical Package for the Social Sciences 28.0 (IBM Corp., Armonk). Continuous data were expressed as mean ± standard deviation and categorical data as frequencies. Normality was assessed using the Shapiro–Wilk test. Baseline differences were analyzed using independent-samples t tests or Mann–Whitney *U* tests for continuous variables and χ^2^ or Fisher exact tests for categorical variables.

To enhance internal validity, propensity score matching was considered; however, given the limited sample size and comparable baseline characteristics, multivariable analysis was used instead. Treatment-related changes in metabolic parameters were evaluated using ANCOVA with adjustment for age, sex, illness duration, and baseline values. Within-group comparisons were performed using paired *t* tests or Wilcoxon signed-rank tests. A two-tailed *P* < .05 was considered statistically significant.

## 3. Results

### 3.1. Baseline characteristics

A total of 107 patients were included in the analysis, with 56 in the WM group and 51 in the integrative medicine group receiving DWD in addition to WM (DWD + WM). There were no statistically significant differences between the 2 groups in terms of demographic and clinical characteristics, including sex distribution, mean age, disease duration, psychiatric diagnosis, and the type or dosage of atypical antipsychotic medication. Similarly, baseline metabolic parameters, including body weight, BMI, waist circumference, blood pressure, fasting plasma glucose, triglycerides, and HDL-C, were comparable between groups (all *P* > .05). These findings suggest that the 2 groups were well balanced at baseline (Table 1).

**Table 1:** Baseline characteristics of patients in the 2 groups.

Variable	Western Medicine (WM) group (n = 56)	DWD + WM group (n = 51)	*P*-value
Sex, n (%)	Male: 33 (58.9%)	Male: 31 (60.8%)	.85
	Female: 23 (41.1%)	Female: 20 (39.2%)	
Age, yr (mean ± SD)	39.7 ± 10.9	40.3 ± 11.1	.74
Disease duration, yr (mean ± SD)	11.3 ± 6.2	11.6 ± 6.8	.81
Primary diagnosis, n (%)	Paranoid schizophrenia: 31 (55.4%)	Paranoid schizophrenia: 30 (58.8%)	.93
	Disorganized: 13 (23.2%)	Disorganized: 12 (23.5%)	
	Other subtypes: 12 (21.4%)	Other subtypes: 9 (17.7%)	
Antipsychotic medication, n (%)	Clozapine: 21 (37.5%)	Clozapine: 19 (37.3%)	.97
	Olanzapine: 19 (33.9%)	Olanzapine: 16 (31.4%)	
	Quetiapine: 10 (17.9%)	Quetiapine: 9 (17.6%)	
	Others: 6 (10.7%)	Others: 7 (13.7%)	
Duration of AAP treatment, mo (mean ± SD)	26.4 ± 11.7	27.1 ± 12.0	.72
Weight, kg (mean ± SD)	74.6 ± 12.4	75.4 ± 13.0	.7
BMI, kg/m^2^ (mean ± SD)	27.2 ± 3.8	27.4 ± 4.0	.76
Waist circumference, cm (mean ± SD)	93.5 ± 8.6	94.0 ± 8.9	.79
Systolic BP, mm Hg (mean ± SD)	132.9 ± 12.5	133.3 ± 12.8	.84
Diastolic BP, mm Hg (mean ± SD)	84.1 ± 8.3	83.8 ± 8.2	.88
Fasting plasma glucose, mmol/L (mean ± SD)	6.1 ± 1.1	6.0 ± 1.2	.66
HbA1c, % (mean ± SD)	6.4 ± 0.7	6.3 ± 0.6	.62
Triglycerides, mmol/L (mean ± SD)	2.15 ± 0.52	2.13 ± 0.48	.81
HDL-C, mmol/L (mean ± SD)	0.98 ± 0.21	0.99 ± 0.22	.9

AAP = atypical antipsychotic, BMI = body mass index, BP = blood pressure, DWD = Dachai Wendan Decoction, HbA1c = glycated haemoglobin, HDL-C = high-density lipoprotein cholesterol, SD = standard deviation, WM = Western Medicine.

### 3.2. Improvements in metabolic syndrome remission, body composition, and blood pressure

At the end of follow-up (8 weeks), the remission rate of MetS, defined according to the IDF criteria, was significantly higher in the integrative medicine group (DWD + WM) compared with the WM group. In the DWD + WM group, 24 patients (47.1%) no longer met the diagnostic criteria for MetS, whereas only 14 patients (25.0%) achieved remission in the WM group (*P* = .02). Both groups demonstrated reductions in body weight and BMI after treatment; however, the magnitude of reduction was greater in the DWD + WM group. The mean decrease in body weight was −3.2 ± 2.1 kg in the DWD + WM group versus −1.5 ± 1.8 kg in the WM group (*P* < .01), while the mean reduction in BMI was −1.1 ± 0.7 kg/m^2^ versus −0.5 ± 0.6 kg/m^2^, respectively (*P* < .01). Similarly, central obesity improved more markedly in the integrative medicine group, with a mean reduction in waist circumference of −4.5 ± 2.8 cm compared with −2.0 ± 2.4 cm in the WM group (*P* < .01). Blood pressure decreased in both groups, but the DWD + WM group exhibited greater improvement. Systolic blood pressure decreased by −9.2 ± 7.5 mm Hg in the DWD + WM group compared with −5.1 ± 6.8 mm Hg in the WM group (*P* = .03), while diastolic blood pressure decreased by −5.4 ± 4.6 mm Hg versus −2.8 ± 4.1 mm Hg, respectively (*P* = .02). These findings indicate that the addition of DWD produced superior improvements in overall MetS remission, weight reduction, central obesity, and blood pressure control compared with WM alone (Table 2).

**Table 2:** Primary outcomes of the 2 groups after 8 wk of treatment.

Outcome	WM group (n = 56)	DWD + WM group (n = 51)	*P*-value
Metabolic syndrome remission, n (%)	12 (21.4%)	22 (43.1%)	.02
Weight change, kg (mean ± SD)	−1.4 ± 1.7	−3.0 ± 2.0	<.01
BMI change, kg/m^2^ (mean ± SD)	−0.4 ± 0.5	−1.0 ± 0.7	<.01
Waist circumference change, cm (mean ± SD)	−1.9 ± 2.3	−4.2 ± 2.7	<.01
Systolic BP change, mm Hg (mean ± SD)	−4.8 ± 6.5	−8.9 ± 7.2	.03
Diastolic BP change, mm Hg (mean ± SD)	−2.6 ± 4.0	−5.1 ± 4.5	.02

Remission defined as no longer fulfilling IDF criteria for metabolic syndrome. Data are presented as mean ± standard deviation (SD) or number (percentage).

BMI = body mass index, BP = blood pressure, DWD = Dachai Wendan Decoction, WM = Western Medicine.

### 3.3. Improvements in lipid profile, glycemic control, and psychiatric symptom stability

After 8 weeks of treatment, both groups demonstrated improvements in lipid metabolism, but the DWD + WM group achieved more pronounced effects. Triglyceride levels decreased by −0.42 ± 0.35 mmol/L in the DWD + WM group compared with −0.21 ± 0.28 mmol/L in the WM group (*P* = .01). Similarly, HDL-C increased significantly in the DWD + WM group (+0.12 ± 0.10 mmol/L) relative to the WM group (+0.05 ± 0.08 mmol/L, *P* = .02). LDL-C and total cholesterol also decreased more in the DWD + WM group (−0.46 ± 0.40 mmol/L and −0.62 ± 0.55 mmol/L, respectively) than in the WM group (−0.25 ± 0.32 mmol/L and −0.38 ± 0.47 mmol/L, *P* < .05 for both). Glycemic control improved in both groups, with fasting plasma glucose decreasing by −0.6 ± 0.5 mmol/L in the DWD + WM group compared with −0.3 ± 0.4 mmol/L in the WM group (*P* = .03). HbA1c also declined more in the DWD + WM group (−0.4 ± 0.3%) than in the WM group (−0.2 ± 0.3%, *P* = .04). Regarding psychiatric stability, no significant differences were observed between the 2 groups. PANSS scores remained stable throughout the treatment period in both groups, and no dosage adjustments of antipsychotic medications were required for symptom control (all *P* > .05). These results indicate that the addition of DWD improved metabolic parameters without compromising psychiatric stability (Table 3).

**Table 3:** Secondary outcomes of the 2 groups after 8 wk of treatment.

Outcome	WM group (n = 56)	DWD + WM group (n = 51)	*P*-value
Triglycerides, mmol/L (Δ)	−0.21 ± 0.28	−0.42 ± 0.35	.01
HDL-C, mmol/L (Δ)	+0.05 ± 0.08	+0.12 ± 0.10	.02
LDL-C, mmol/L (Δ)	−0.25 ± 0.32	−0.46 ± 0.40	.04
Total cholesterol, mmol/L (Δ)	−0.38 ± 0.47	−0.62 ± 0.55	.03
Fasting plasma glucose, mmol/L (Δ)	−0.3 ± 0.4	−0.6 ± 0.5	.03
HbA1c, % (Δ)	−0.2 ± 0.3	−0.4 ± 0.3	.04
PANSS score (Δ)	−0.4 ± 1.2	−0.5 ± 1.3	.78

Data are presented as mean change (Δ, mean ± SD) from baseline to week 8.

DWD = Dachai Wendan Decoction, HbA1c = glycated haemoglobin, HDL-C = high-density lipoprotein cholesterol, LDL-C = low-density lipoprotein cholesterol, PANSS = Positive and Negative Syndrome Scale, SD = standard deviation, WM = Western Medicine.

### 3.4. Safety and adverse events

During the 8-week treatment period, both groups demonstrated good overall safety and tolerability. The most frequently reported adverse events were mild gastrointestinal discomfort (such as nausea, bloating, or diarrhea), mild elevations in liver transaminases, and transient fatigue. No severe adverse reactions, including hepatotoxicity, nephrotoxicity, or allergic reactions requiring medical intervention, were observed. The overall incidence of adverse events was 16.1% (9/56) in the WM group and 13.7% (7/51) in the DWD + WM group, with no statistically significant difference between groups (*P* = .72). Among patients receiving DWD, 3 cases of mild gastrointestinal discomfort and 2 cases of mild transient liver enzyme elevation were recorded, all of which resolved spontaneously or after symptomatic management without discontinuation of therapy. Treatment adherence was high in both groups, with >85% of patients completing the 8-week protocol. No patients discontinued therapy due to adverse reactions. These findings suggest that the addition of DWD was generally safe and well tolerated, without increasing the risk of adverse events compared with WM alone (Table 4).

**Table 4:** Safety and adverse events during 8 wk of treatment.

Adverse events	WM group (n = 56)	DWD + WM group (n = 51)	*P*-value
Gastrointestinal discomfort, n (%)	4 (7.1%)	3 (5.9%)	.82
Mild transaminase elevation, n (%)	3 (5.4%)	2 (3.9%)	.71
Renal function abnormality, n (%)	1 (1.8%)	1 (2.0%)	.95
Allergic reaction (mild), n (%)	1 (1.8%)	1 (2.0%)	.95
Total adverse events, n (%)	9 (16.1%)	7 (13.7%)	.72
Discontinuation due to adverse events	0	0	–
Treatment adherence ≥ 80%, n (%)	54 (96.4%)	49 (96.1%)	.94

Data are presented as number (percentage).

DWD = Dachai Wendan Decoction, WM = Western Medicine.

## 4. Discussion

This retrospective analysis demonstrates that adding DWD to standard Western management for atypical-antipsychotic–associated MetS was associated with higher MetS remission at 8 weeks and greater improvements in body weight, BMI, waist circumference, and blood pressure compared with WM alone, without compromising psychiatric stability or safety. The consistency of effects across anthropometric, hemodynamic, lipid, and glycemic domains suggests a broad metabolic benefit. Importantly, PANSS scores remained stable and antipsychotic doses were not escalated, indicating that metabolic gains were not achieved at the expense of psychiatric control. Adverse events were infrequent, mild, and comparable between groups, and treatment adherence exceeded 85% in both arms.

Several mechanisms may account for the observed differences. Central obesity and insulin resistance are key features of antipsychotic-induced metabolic dysfunction, mediated by effects on appetite regulation, energy expenditure, and peripheral insulin signaling. DWD is classically formulated to “resolve phlegm-heat” and “harmonize the gallbladder–stomach axis.” From a biomedical perspective, component herbs in Wendan-based prescriptions have been reported to influence lipid handling, bile acid flow, gastric motility, and low-grade inflammation, pathways relevant to antipsychotic-related weight gain and dysmetabolism. The larger reductions in waist circumference observed here align with a potential effect on visceral adiposity, which has disproportionate cardiometabolic impact relative to overall mass.^[7,8]^ The concomitant declines in systolic and diastolic blood pressure may reflect weight-mediated effects and improved endothelial or autonomic function; the absence of signal for hepatotoxicity or nephrotoxicity supports acceptable near-term tolerability of DWD when co-administered with psychotropics and cardio-metabolic agents under routine care.

The lipid and glycemic findings extend the anthropometric results. Compared with WM alone, DWD + WM was associated with larger decreases in triglycerides and total/LDL cholesterol, and a greater rise in HDL-C. Fasting glucose and HbA1c also improved more in the DWD + WM arm over 8 weeks. These short-term biochemical changes are directionally concordant with the anthropometric and blood pressure responses and together are likely to contribute to the higher MetS remission rate defined by IDF criteria. Psychiatric stability across groups suggests that the metabolic improvements are not attributable to differential antipsychotic de-intensification.^[9,10]^

Contemporary guidance emphasizes systematic metabolic monitoring and structured lifestyle interventions for all patients receiving antipsychotics, with pharmacologic add-ons as needed. A 2023 narrative review underscored the consistent efficacy of aerobic exercise and dietetic counseling for antipsychotic-induced MetS, while noting that only a few pharmacological strategies demonstrate reliable benefit in this population.^[11]^ Our WM protocol incorporated guideline-concordant lifestyle and cardiometabolic therapy, consistent with recommendations that prioritize baseline and interval screening (e.g., weight/BMI and waist circumference at baseline, at 4 months after antipsychotic initiation or change, and annually thereafter). Metformin remains the most consistently supported pharmacologic option for antipsychotic-induced weight gain and dysglycemia, with growing calls for earlier and broader use; benefits extend to patients on clozapine and those initiating second-generation antipsychotics.^[12]^ GLP-1 receptor agonists show promise in cases refractory to metformin, though current evidence in antipsychotic-treated cohorts is limited to small trials and case series.^[13]^ Within this context, our data suggest that DWD as an adjunct to standard WM may augment weight, waist, lipid, and glycemic responses over 8 weeks. Although direct head-to-head comparisons with metformin or GLP-1 agents were not performed here, the incremental effects observed on top of routine care are clinically relevant.

Evidence for Wendan-based prescriptions in schizophrenia largely addresses psychiatric outcomes and antipsychotic tolerability rather than metabolic endpoints. A Cochrane review reported that Wendan decoction might improve short-term global mental state and reduce extrapyramidal symptoms when combined with antipsychotics, but did not demonstrate weight benefit; the metabolic dataset was sparse.^[14]^ In contrast, outside schizophrenia, recent syntheses of TCM combined with WM for MetS (non-psychiatric populations) indicate superior improvements in composite MetS remission and individual metabolic parameters versus WM alone, although heterogeneity and risk of bias vary across trials.^[15]^ Emerging preclinical and clinical signals from Wendan-derivative prescriptions (e.g., Huanglian Wendan) report effects on obesity, insulin sensitivity, and dyslipidemia, supporting plausibility for metabolic modulation, but these data are not specific to antipsychotic-treated patients and require confirmation.^[16]^ Taken together, our findings align with the broader trend that multimodal strategies – lifestyle, foundational pharmacotherapy (e.g., metformin), and, potentially, targeted TCM adjuncts – may yield additive metabolic benefits in antipsychotic-treated patients when psychiatric regimens are kept stable and safety is monitored.

The higher MetS remission with DWD + WM likely reflects concurrent effects on central adiposity and triglyceride-rich lipoprotein metabolism, combined with modest improvements in fasting glycemia and blood pressure. From a clinical perspective, even small short-term changes in waist circumference and triglycerides can shift patients below diagnostic thresholds for MetS, thereby reducing near-term cardiometabolic risk profiling. The absence of psychiatric destabilization indicates feasibility of introducing DWD without altering antipsychotic therapy, a key practical consideration in real-world schizophrenia care. These results support DWD as a pragmatic adjunct to routine cardiometabolic management for patients receiving AAPs. In settings where structured lifestyle counseling and metformin or statins are standard, adding DWD may increase the probability of achieving short-term MetS remission and yield greater reductions in weight, waist circumference, triglycerides, and blood pressure without compromising psychiatric stability. Implementation should include baseline and interval monitoring of anthropometrics, fasting lipids/glucose, and liver/renal function, consistent with current metabolic surveillance guidance for antipsychotic-treated populations.

Strengths include: a clearly defined cohort of antipsychotic-treated patients with MetS, prespecified and clinically relevant primary outcomes (IDF-defined MetS remission; anthropometric and blood pressure changes), concurrent evaluation of lipid and glycemic endpoints, and systematic safety capture showing comparable adverse event rates and high adherence across groups. Psychiatric stability and medication dosing constancy reduce confounding by indication related to antipsychotic changes. Limitations warrant caution. First, the retrospective design is susceptible to selection bias, unmeasured confounding, and information bias; although baseline comparability was demonstrated, residual confounding cannot be excluded. Second, the 8-week horizon limits evaluation of durability; antipsychotic-induced metabolic changes evolve over months, and long-term cardiometabolic outcomes were not assessed. Third, individualized modifications to herbal components, while reflective of practice, introduce heterogeneity; dose–response relationships and pharmacokinetic interactions with psychotropics were not examined. Fourth, we did not formally standardize lifestyle intensity, and although adherence proxies were high, objective measures (e.g., digital activity or dietary logs) were not available. Fifth, safety conclusions are constrained by sample size and duration; rare hepatic or renal adverse events may emerge with longer exposure. Finally, generalizability beyond similar integrative-medicine settings requires confirmation in randomized, adequately powered trials with blinded outcome assessment and protocolized co-interventions.

## 5. Conclusions

In summary, the addition of DWD to standard WM significantly improved short-term metabolic outcomes in patients with AIMS. Compared with WM alone, combined therapy resulted in higher remission rates, greater reductions in weight, BMI, waist circumference, and blood pressure, as well as more favorable changes in lipid and glycemic profiles, without compromising psychiatric stability or safety. These findings support its role as a safe and effective adjunctive strategy.

## Author contributions

**Conceptualization:** Yuze Wu, Xia Zhao, Jiefeng Cui, Menghan Lv, Bo Hu, Junran Zheng, Fude Yang, Shaoxiao Yan.

**Data curation:** Yuze Wu, Xia Zhao, Jiefeng Cui, Junran Zheng, Fude Yang, Shaoxiao Yan.

**Formal analysis:** Yuze Wu, Xia Zhao, Jiefeng Cui, Menghan Lv, Bo Hu, Junran Zheng, Fude Yang, Shaoxiao Yan.

**Funding acquisition:** Yuze Wu, Xia Zhao, Bo Hu, Junran Zheng, Fude Yang, Shaoxiao Yan.

**Investigation:** Yuze Wu, Fude Yang, Shaoxiao Yan.

**Writing – original draft:** Yuze Wu, Xia Zhao, Menghan Lv, Chunyan Xu, Fude Yang, Shaoxiao Yan.

**Writing – review & editing:** Yuze Wu, Xia Zhao, Menghan Lv, Chunyan Xu, Fude Yang, Shaoxiao Yan.
